# 20(S)-ginsenoside Rh2 as agent for the treatment of LMN-CRC via regulating epithelial–mesenchymal transition

**DOI:** 10.1042/BSR20191507

**Published:** 2020-03-25

**Authors:** Yihang Yuan, Jue Wang, Ming Xu, Yunpeng Zhang, Zhiqiang Wang, Leilei Liang, Peng Sun

**Affiliations:** Department of General Surgery, Tongren Hospital, Shanghai Jiao Tong University School of Medicine, 1111 XianXia Road, Shanghai 200336, China

**Keywords:** 20(S)-ginsenoside Rh2, EMT, LMN-CRC

## Abstract

The lymph node metastasis of colorectal cancer (LMN-CRC) seriously threatens the prognosis of patients. Chemotherapy, as the most common treatment, results in severe bone marrow suppression. 20(S)-ginsenoside Rh2 (SGRh2), a major effective constituent of ginseng, has demonstrated therapeutic effects on a variety of diseases, including some tumours. SGRh2 treatment had no effect on other organs. Therefore, ginsenosides are considered a safe and effective antineoplastic drug. However, the effects of SGRh2 on LMN-CRC remain unknown. The present study investigated the potential effect of SGRh2 on LMN-CRC *in vitro* and *in vivo*. SW480 and CoLo205 cell lines were treated with SGRh2. SGRh2 dose-dependently decreased CRC cell proliferation by CCK-8, colony formation and Edu assays. The Transwell and scratch assays revealed that SGRh2 inhibits the migratory and invasive abilities of CRC cells in a dose-dependent manner. Furthermore, the results of Western blotting revealed that SGRh2 decreased the expression of matrix metalloproteinase (MMP)-2 and MMP9. In terms of the underlying mechanisms, SGRh2 regulates CRC metastasis by affecting epithelial–mesenchymal transition (EMT), which significantly up-regulated epithelial biomarkers (E-cadherin) and down-regulated mesenchymal biomarkers (N-cadherin and vimentin) and EMT transcriptional factors (Smad-3, Snail-1, and Twist-1). *In vivo*, SGRh2 significantly inhibited LMN-CRC without affecting other normal organs. Immunohistochemical results showed that SGRh2 treats LMN-CRC by regulating EMT. These results demonstrate that SGRh2 has therapeutic potential for LMN-CRC.

## Introduction

According to the statistics of the 2018 Global Cancer Annual Report, colorectal cancer (CRC) is the third leading cause of death [1]. The lymph node metastasis of colorectal cancer (LMN-CRC) is the most common route of CRC metastasis and an important factor affecting the prognosis of CRC patients [2]. The NCCN guidelines indicate that patients with lymph node metastases need to undergo further chemotherapy after surgery. The severe bone marrow depression caused by chemotherapy often causes patients to miss the treatment opportunity and delay treatment [3]. Therefore, safe and effective therapeutic strategies for LMN-CRC are needed.

Ginseng has long been used as a medicinal herb in China for more than 2000 years. Many studies have shown that ginsengs have preventive and therapeutic roles for cancer and play a good complementary role in cancer treatment. Ginsenosides are the major active components of ginseng extracts and have been extensively investigated and emphasized in cancer chemoprevention and therapeutics without influencing normal cells [4]. 20(S)-ginsenoside Rh2 (SGRh2), the most well-known compound of ginsenosides, has demonstrated therapeutic effects on a variety of diseases, including some tumours [5,6]. Recent studies have shown that SGRh2 induces apoptosis and paraptosis-like cell death in CRC cells through the activation of p53 [7]. The exact molecular mechanisms of SGRh2 on LMN-CRC have still not been clarified.

Epithelial–mesenchymal transition (EMT) is a process in which epithelial cells acquire mesenchymal features and plays an important role in tumourigenesis and metastasis [8,9]. During the EMT process, carcinomas often lose epithelial markers (E-cadherin) and express EMT markers (N-cadherin and vimentin) at the invasive front [10,11]. In addition, the EMT process is orchestrated by a set of transcription factors (EMT-TFs), including Twist and Snail [12,13]. The expression of EMT-TFs correlates with poor clinical outcomes in cholangiocarcinoma, gastric cancer and breast cancer [14–16]. However, whether and how EMT stimulates LMN-CRC tumourigenesis remains largely unknown.

In the present study, we explored the potential effect of SGRh2 on LMN-CRC *in vitro* and *in vivo*. Moreover, we found that the effect of SGRh2 was mediated by EMT and EMT-TFs. The data herein clearly showed that SGRh2 triggers EMT and EMT-TFs to cause cancer cell proliferation, migration and invasion. Our results provide evidence supporting SGRh2 as a novel anticancer agent for the treatment of LMN-CRC.

## Materials and methods

### Cell culture and 20(S)-ginsenoside Rh2

The human CRC cell lines SW480 (Cat.No:SCSP-5033) and CoLo205 (Cat.No:TCHu102) were obtained from the Shanghai Cell Bank of the Chinese Academy of Sciences (Shanghai, China). SW480 cells were cultured in DMEM, and CoLo205 cells were cultured in RPMI-1640 supplemented with 10% foetal bovine serum (Cat.No:10100147, Gibco, Life Technologies, U.S.A.). The cells were incubated at 37°C and 5% CO_2_. 20(S)-ginsenoside Rh2 (SGRh2) was purchased from Solarbio (Cat.No:YZ-111748, Beijing, China).

### Cell proliferation assay

Cell viability was assessed colorimetrically using a cell counting kit-8 (CCK-8, Cat.No:C0038, Beyotime, Shanghai, China). Human CRC cells (1 × 10^5^) were seeded in each well of a 96-well plate and incubated for 24, 48 and 72 h prior to treatment with different dosages of SGRh2 or vehicle. After treatment, 10 μl of CCK-8 solution was added into each well and incubated for an additional 2 h. The absorbance value at 450 nm was read using a microplate reader (Bio-Rad, CA, U.S.A.).

### Colony formation assay

With respect to the colony formation assay, 1 × 10^3^ cells were seeded in six-well plates. The cells were mixed and then cultured for 1 week in culture medium with 10% FBS (Cat.No:10100147, Gibco, Life Technologies, U.S.A.). Clusters containing ≥30 cells were counted as a single colony.

### Ethynyl deoxyuridine (Edu) analysis

The cells were cultured in 96-well plates at a density of 4 × 10^4^ cells/well. Forty-eight hours after transfection, 20 μM Edu labelling media (Cat.No:KGA337-500, KeyGEN BioTECH, Nanjing, China) was added to the 96-well plates, which were then incubated for 2 h at 37°C and 5% CO_2_. After treatment with 4% paraformaldehyde and 0.5% Triton X-100, the cells were stained with anti-Edu working solution. The percentage of Edu-positive cells was calculated after fluorescence microscopy analysis.

### Cell migration and invasion assays

The cell invasion and migration abilities of CRC cells were evaluated by a Transwell assay (Cat.No:3422, Corning, NY, U.S.A.). Cells were treated with SGRh2 for 24 h and then harvested. A total of 5 × 10^4^ cells in serum-free medium were placed into the upper chamber of an insert of the Transwell assay, while medium supplemented with 10% FBS was added into the lower chamber for chemo-attraction. Transwell membrane filters were used for the migration assays and Transwell membrane filters coated with Matrigel (Cat.No: 354234, Corning, NY, U.S.A.) were used for the invasion assays. After incubation at 37°C for 24 h, the cells were stained with methanol and 0.1% Crystal Violet, imaged and counted using an Olympus microscope (Tokyo, Japan).

### Wound healing assay

The cells were seeded in a 12-well plate to form a monolayer one day before the assay. The migration path of cells was tracked at 0 and 24 h using an Olympus microscope (10 × 10). The results of the experiments were analyzed by ipp 6.0 software (Media Cybernetics, Bethesda, MD, U.S.A.).

### Western blot assay and antibodies

Total protein was isolated from the cells with RIPA lysis buffer (Cat.No:KGP702, KeyGEN, Nanjing, China). Twenty micrograms of protein per well was electrophoresed in 10% SDS-PAGE gels and then transferred to polyvinylidene difluoride membranes (Cat.No:ISEQ00010, Millipore, Bedford, MA, U.S.A.). The membranes were incubated with different primary antibodies, including E-cadherin (dilution 1:3000, Cat.No:ab76055, Abcam, the U.K.), N-cadherin (dilution 1:3000, Cat.No:ab76057, Abcam, the U.K.), Vimentin (dilution 1:3000, Cat.No:ab92547, Abcam, the U.K.), Snail-1 (dilution 1:3000, Cat.No:ab53519, Abcam, the U.K.), Twist-1 (dilution 1:3000, Cat.No:ab175430, Abcam, the U.K.), Smad-3 (dilution 1:3000, Cat.No:ab40854, Abcam, the U.K.), MMP2 (dilution 1:3000, Cat.No:ab37150, Abcam, the U.K.), MMP9 (dilution 1:3000, Cat.No:ab58803, Abcam, the U.K.) and GAPDH (dilution 1:30000, Cat.No:ab181602, Abcam, the U.K.), at 4°C overnight and then incubated with secondary antibodies (dilution 1:30000, Cat.No:ab97051, Abcam, the U.K.). The bands were visualized with ECL reagent (Cat.No:32134, Thermo Fisher Scientific, U.S.A.) and GAPDH was used as the loading control.

### Immunofluorescence assay

Cells were plated on glass slides in 24-well plates and subsequently treated with SGRh2 for 24 h. The cells were then fixed in 4% paraformaldehyde, permeabilized with 0.5% Triton X-100, and incubated overnight with the indicated primary antibody: E-cadherin (dilution 1:100, Cat.No:ab76055, Abcam, the U.K.), N-cadherin (dilution 1:100, Cat.No:ab76057, Abcam, the U.K.), Vimentin (dilution 1:100, Cat.No:ab92547, Abcam, the U.K.). Afterwards, the cells were incubated with DAPI (Cat.No:P36931, Thermo Fisher Scientific, U.S.A.). The images were obtained with a fluorescence microscope (OLYMPUS).

### Immunohistochemistry staining

Xenograft tumours were processed into paraffin-embedded sections. The slides were dried, deparaffinized and rehydrated. Then, the slides were immersed in 3% hydrogen peroxide and labelled with Ki67 (dilution 1:200, Cat.No:ab16667, Abcam, the U.K.), E-cadherin (dilution 1:200, Cat.No:ab76055, Abcam, the U.K.), N-cadherin (dilution 1:200, Cat.No:ab76057, Abcam, the U.K.), Vimentin (dilution 1:200, Cat.No:ab92547, Abcam, the U.K.) antibodies overnight. The slides were stained with the secondary streptavidin-horseradish peroxidase-conjugated antibody (Cat.No:SP0041, Solarbio, Beijing, China). The slides were then counterstained with haematoxylin for 30 s and coverslipped.

### Tumourigenicity assay *in vivo*

Female BALB/c nude mice (≈20 g) were provided by Shanghai Laboratory Animal Center (Chinese Academy of Sciences, Shanghai, China). Animal experiments were performed in the Animal Facility of Shanghai Jiao Tong University School of Medicine. The animal experiment designed in the present study was approved by the ethical committee of Shanghai Jiao Tong University School of Medicine (SJTU-SM). A cell suspension of CoLo205 (2 × 10^6^ in 100 µl) labelled with luciferase was injected into the submucosa of the terminal caecum wall of the nude mice. The metastasis of lymph nodes around the tumour in the animal model was observed from 4 weeks by the Caliper IVIS Lumina II System. Then, SGRh2 (5 mg/kg) or vehicle was injected three times a week for 4 weeks, and the tumour tissues from mice were embedded in a paraffin block.

### Immunohistochemistry staining

Xenograft tumours were processed into paraffin-embedded sections. The slides were dried, deparaffinized and rehydrated. Then, the slides were immersed in 3% hydrogen peroxide and labelled with Ki67 (dilution 1:200, Cat.No:ab16667, Abcam, the U.K.), E-cadherin (dilution 1:200, Cat.No:ab76055, Abcam, the U.K.), N-cadherin (dilution 1:200, Cat.No:ab76057, Abcam, the U.K.), Vimentin (dilution 1:200, Cat.No:ab92547, Abcam, the U.K.) antibodies overnight. The slides were stained with the secondary streptavidin-horseradish peroxidase-conjugated antibody (Cat.No:SP0041, Solarbio, Beijing, China). The slides were then counterstained with haematoxylin for 30 s and cover slipped.

### Statistical analysis

Student’s *t*-test was used to examine the differences between groups. A value of *P* < 0.05 was regarded as statistically significant. All calculations were performed using SPSS software version 13.0.

## Results

### 20(S)-ginsenoside Rh2 suppresses the viability and proliferation ability of CRC cells

**Figure 1:**
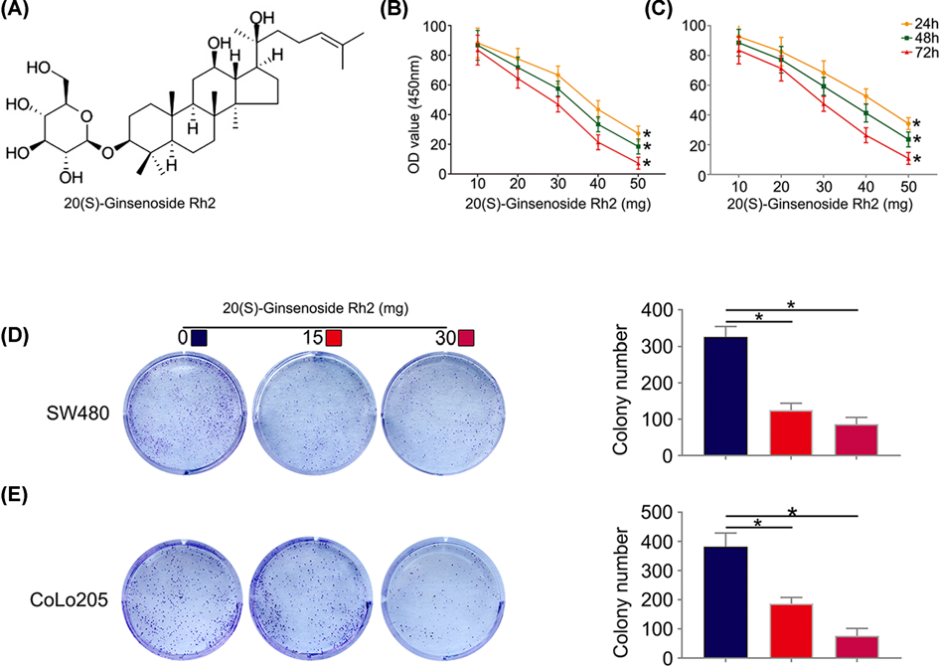
Dose-dependent antiproliferative effects of SGRh2 on CRC cells The chemical structure of SGRh2 (**A**). SW480 (**B**) and CoLo205 (**C**) cells after treatment with SGRh2 (0, 10, 20, 30, 40 or 50 mg/l) for 24, 48 or 72 h. SGRh2 suppressed the proliferation of both cell lines in a dose-dependent manner. SGRh2 inhibited the colony formation of SW480 (**D**) and CoLo205 (**E**) cell lines in a dose-dependent manner; *P<0.05.

**Figure 2:**
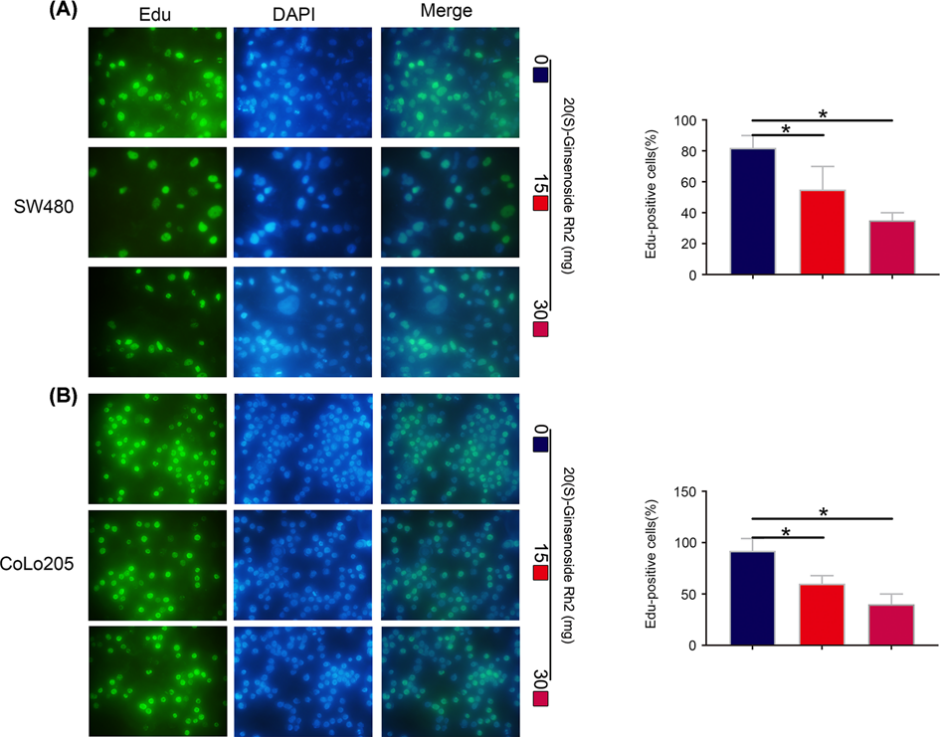
SGRh2 suppresses the proliferation ability of CRC cells by Edu assay An Edu assay showed that SGRh2 suppresses the proliferation ability of SW480 (**A**) and CoLo205 (**B**) cells in a dose-dependent manner; *P<0.05.

The structure of SGRh2 was drawn using ChemDraw 14.0 and is shown in Figure 1A. The effects of SGRh2 on CRC cell viability were investigated by CCK-8 assay. SW480 and CoLo205 cells were treated with increasing concentrations of SGRh2 (0, 10, 20, 30, 40 and 50 µM) for 24, 48 and 72 h. SGRh2 suppressed the viability and proliferation of both SW480 and CoLo205 cells in a dose-dependent manner (Figure 1B,C). Moreover, colony formation and Edu assays also showed that SGRh2 suppressed the proliferation ability of CRC cells (Figures 1D,E and 2A,B). These results indicate that treatment with SGRh2 can inhibit the proliferation of CRC cells.

### 20(S)-ginsenoside Rh2 inhibited the invasiveness and migration of CRC cells

**Figure 3:**
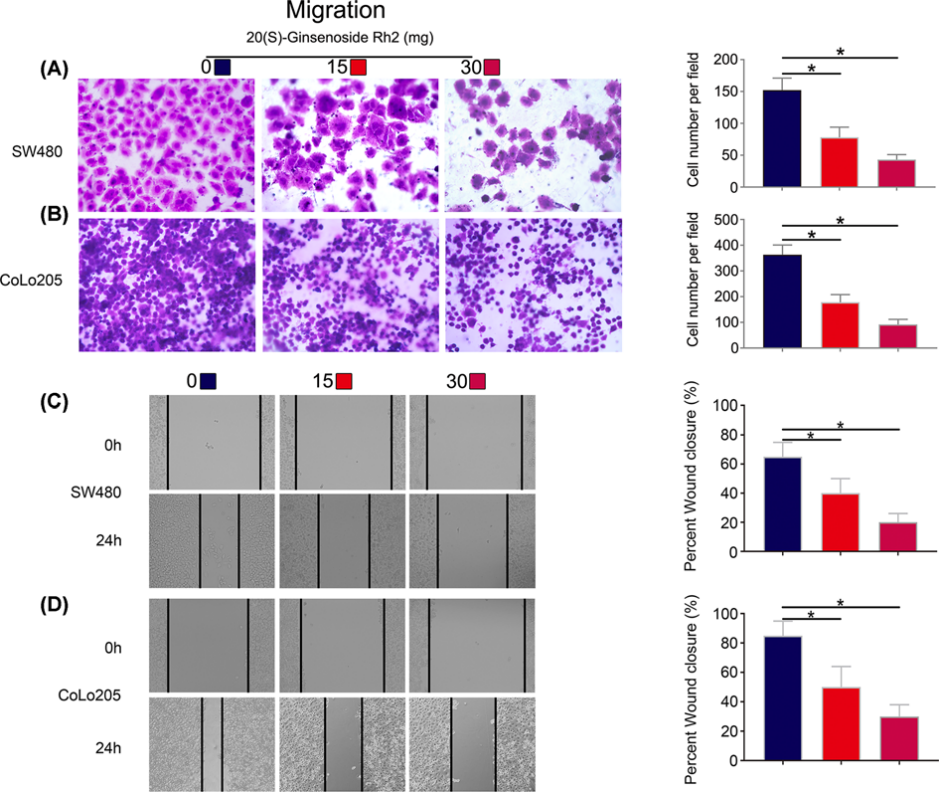
SGRh2 inhibited the migration ability of CRC cells The cells were treated with SGRh2 for 24 h. The images show that SGRh2 inhibited the migration (**A** and **C**) of SW480 cells onto the underside of the transwell inserts and tested by scratch wound healing assay. Similarly, SGRh2 inhibited the migration (**B** and **D**) of CoLo205 cells; *P<0.05.

**Figure 4:**
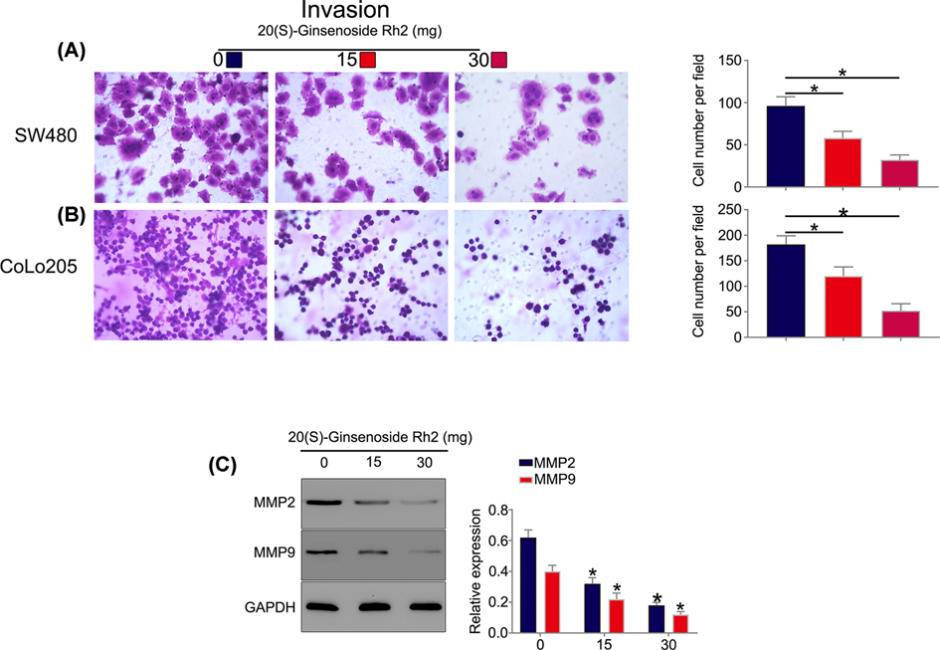
SGRh2 attenuated the invasion and expression of MMP-2 and MMP-9 in CRC cells Cell invasion was tested by transwell assay. The invasion capacity was downregulated in SW480 (**A**) and CoLo205 (**B**) cells. MMP-2 and MMP-9 are important enzymes that participate in tumour invasion. The results showed that SGRh2 dose-dependently attenuated the expression of MMP-2 and MMP-9 in CRC cells (**C**); *P<0.05.

To examine the effect of SGRh2 on cell invasion and migration, Transwell and wound healing assays were performed. Transwell (Figure 3A,B) and wound healing (Figure 3C,D) assay results showed that the cell migration efficiency of SW480 and CoLo205 cells was significantly suppressed after SGRh2 treatment compared with that of the control group. Furthermore, as shown in Figure 4A,B, cell migration was down-regulated in the presence of SGRh2. In addition, we further explored the changes in the expression of MMP-2 and MMP-9 in CRC cells by Western blotting that were associated with cell invasion and migration [17]. The results showed that SGRh2 dose-dependently attenuated the expression of MMP-2 and MMP-9 in CRC cells (Figure 4C), indicating that SGRh2 affected the migration and invasion of CRC cells through a downregulation of MMP-2 and MMP-9 expression.

### 20(S)-ginsenoside Rh2 suppresses EMT in CRC cells

**Figure 5:**
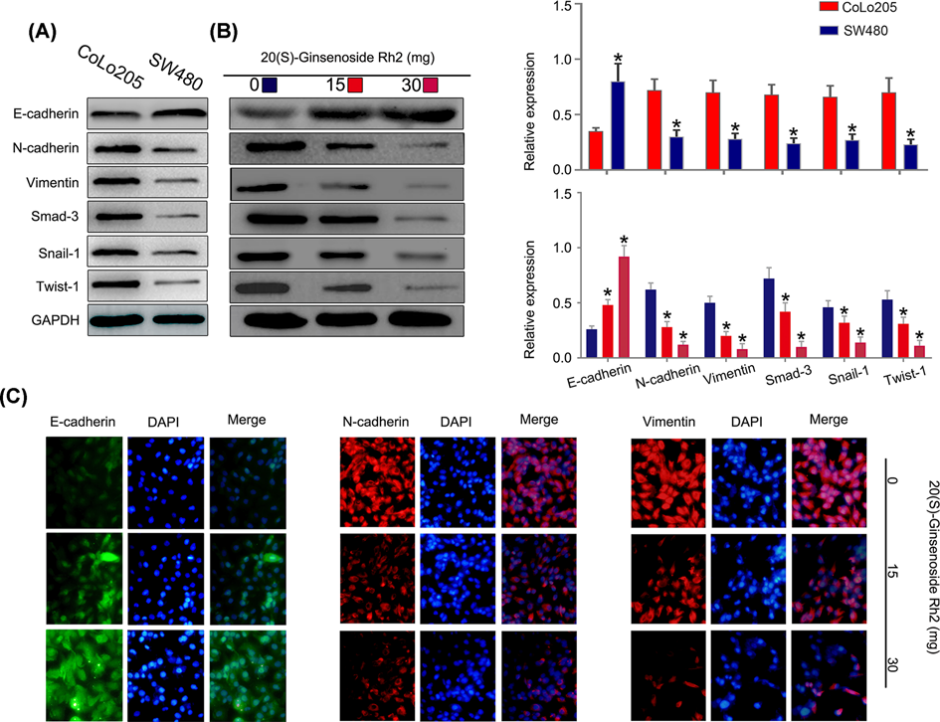
SGRh2 inhibited the expression of EMT biomarkers and EMT-TFs in CRC cells The expression of E-cadherin was enhanced, and the expression of N-cadherin, vimentin, Smad-3, Snail-1 and Twist-1 was reduced in SW480 cells compared with CoLo205 cells (**A**). The protein expression levels of E-cadherin were increased and those of N-cadherin, vimentin, Smad-3, Snail-1 and Twist-1 were decreased by SGRh2 treatment in CoLo205 cells (**B**). Immunofluorescent analysis showed an increased expression of E-cadherin and a decreased expression of N-cadherin and vimentin after treatment with SGRh2 in CoLo205 cells (**C**); *P<05.

To study the potential molecular mechanism underlying the cancer inhibitory effects of SGRh2, we examined the ability of SGRh2 to regulate EMT and EMT-TFs in CRC cells. First, the expression of epithelial markers (E-cadherin), EMT markers (N-cadherin and vimentin) and EMT-TFs (Smad-3, Snail-1 and Twist-1) in CRC cells was measured by Western blotting. As shown in Figure 5A, the expression of E-cadherin was enhanced, and the expression of N-cadherin, vimentin, Smad-3, Snail-1 and Twist-1 were reduced in SW480 cells compared with CoLo205 cells. In addition, we assessed the influence of SGRh2 on CRC treatment. The protein expression levels of E-cadherin were increased, and those of N-cadherin, vimentin, Smad-3, Snail-1 and Twist-1 were decreased by SGRh2 at high concentrations of 15 or 30 mg/l in CoLo205 cells (Figure 5B). Consistently, immunofluorescent analysis showed an increased expression of E-cadherin and a decreased expression of N-cadherin and vimentin after treatment with 15 or 30 mg/l SGRh2 in CoLo205 cells (Figure 5C). The results clearly indicate that SGRh2 could directly bind with E-cadherin, N-cadherin, vimentin, Smad-3, Snail-1 and Twist-1 protein. Therefore, these results show that SGRh2 could directly bind to EMT markers and inhibit EMT-TF activity.

### 20(S)-ginsenoside Rh2 treatment in LMN-CRC *in vivo*

**Figure 6:**
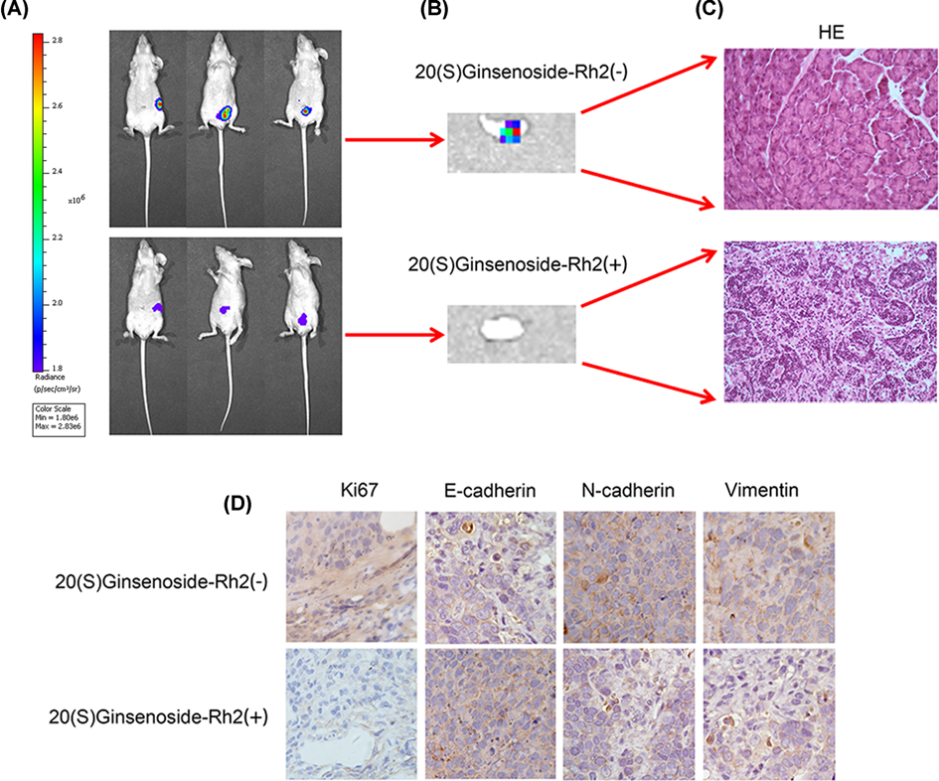
20(S)-ginsenoside Rh2 treatment in LMN-CRC *in vivo* The data indicate that 5 mg/kg of SGRh2 significantly suppressed the lymph node metastasis of colorectal cancer and tumour growth relative to those of the vehicle groups (**A** and** B**). HE staining showed that there were no atypical cells in the lymph nodes after SGRh2 treatment (**C**). HE staining showed that SGRh2 did not inflict any heart, liver, spleen, lung or kidney damage in the mice. Immunostaining analysis showed that E-cadherin protein was increased and Ki67, N-cadherin, and vimentin protein was reduced in the SGRh2-administered group (**D**); *P<0.05.

To further explore the therapeutic effect of ginsenosides *in vivo*, we constructed an LMN-CRC nude mouse model. The mice were divided into a control and an SGRh2-administered group. The mice were then administered vehicle or SGRh2 by injection of 5 mg/kg three times a week for 4 weeks. The data indicate that 5 mg/kg of SGRh2 significantly suppressed the lymph node metastasis of colorectal cancer and tumour growth relative to those of the vehicle groups (Figure 6A,B). HE staining showed that there were no atypical cells in the lymph nodes after SGRh2 treatment (Figure 6C). Furthermore, HE staining showed that SGRh2 did not inflict any heart, liver, spleen, lung or kidney damage in the mouse organs (Figure 6D). To elucidate the mechanism of SGRh2 treatment *in vivo*, immunostaining analyses of Ki67, E-cadherin, N-cadherin and vimentin were performed in the harvested tumour tissues. In the SGRh2-administered group, E-cadherin protein was increased and Ki67, N-cadherin, and vimentin protein was reduced (Figure 6D). Altogether, these findings confirmed that SGRh2 has therapeutic potential for LMN-CRC *in vivo*. The immunohistochemical results showed that SGRh2 treats LMN-CRC by regulating EMT.

## Discussion

Colorectal cancer (CRC) remains the third leading cause of death worldwide, largely due to metastasis. The lymph node metastasis of colorectal cancer (LMN-CRC) is the most common route of CRC metastasis [18]. A better understanding of the biology of LMN-CRC is essential for establishing effective treatment methods. EMT is a developmental process whereby stationary, adherent cells acquire the ability to migrate. The importance of EMT function in cancer metastasis has been widely recognized [19]. EMT leads to cell-mediated metastasis in many types of cancers, including breast cancer [20], pancreatic cancer [21] and lung cancer [22]. However, the relationship between EMT and LMN-CRC has not been reported. For the first time, we observed that EMT contributes to LMN-CRC in CRC cells (Figure 5A). In addition, EMT continues to be an attractive target for cancer therapy [23]. In a previous study, ginsenosides demonstrated therapeutic effects on cancer metastasis [24], including metastatic melanoma [25] breast cancer metastasis [26] and thyroid cancer metastasis [27]. GRh2, a well-known compound of ginsenosides, also has a therapeutic effect on cancer metastasis. For example, GRh2 can inhibit the metastasis of glioblastoma multiforme [28]. However, SGRh2 has not been reported for the treatment of LMN-CRC.

In the present study, we investigated whether SGRh2 has a therapeutic effect on LMN-CRC *in vitro* and *in vivo*. Our data from the functional study confirmed that SGRh2 can reduce the proliferation, migration and invasion ability of CRC cells *in vitro.* Our results are consistent with the results of Han et al. [29]. These results suggest that SGRh2 has therapeutic potential for LMN-CRC and that it may be a novel anticancer agent for patients with LMN-CRC. Furthermore, mechanistic experiments showed that the expression of E-cadherin was enhanced, and the expression of EMT markers (N-cadherin and vimentin) and EMT-TFs (Smad-3, Snail-1 and Twist-1) were reduced in SW480 cells compared with CoLo205 cells by Western blot assay. The protein expression levels of E-cadherin were increased and those of EMT markers and EMT-TFs were decreased by SGRh2 at high concentrations of 15 or 30 mg/l in CoLo205 cells. Consistently, immunofluorescent analysis showed an increased expression of E-cadherin and a decreased expression of EMT markers after treatment with SGRh2 in CoLo205 cells. *In vivo*, the results showed that SGRh2 significantly suppressed LMN-CRC and tumour growth. Furthermore, HE staining showed that SGRh2 did not inflict any heart, liver, spleen, lung or kidney damage in the mouse organs. In the SGRh2-administered group, the immunostaining analysis of xenografted tumour tissues revealed that E-cadherin protein was increased and Ki67, N-cadherin, and Vimentin protein was reduced. Taken together, these data suggest that EMT is involved in LMN-CRC and that SGRh2 significantly suppresses LMN-CRC by regulating EMT.

For the first time, we identified that EMT plays an important role in LMN-CRC and that EMT can be used as an effective target for SGRh2 in the treatment of LMN-CRC *in vitro* and *in vivo*. Furthermore, the *in vivo* studies of a xenograft mouse model indicated that SGRh2 does not influence other normal organs when treating cancer. These results are novel findings, and SGRh2 may be a promising therapeutic candidate for LMN-CRC patients. However, only two CRC cell lines were used to verify the SGRh2 treatment in the present study. In our further investigations, additional CRC cell lines are still needed to explore the treatment of SGRh2 and provide a better understanding of the mechanisms for the beneficial effects of SGRh2 in human health.

In conclusion, the present study identified that EMT is a direct and important target of SGRh2 for the suppression of LMN-CRC *in vitro* and *in vivo*. To our knowledge, our study is the first to show a role for EMT and EMT-TFs in LMN-CRC that could be regulated by the application of SGRh2. These studies provide a better understanding of the mechanisms for the beneficial effects of SGRh2 in LMN-CRC, and SGRh2 may possess a therapeutic potential for LMN-CRC by inhibiting EMT and EMT-TFs.

## Abbreviations

CRC: colorectal cancer
EMT: epithelial–mesenchymal transition
LMN: lymph node metastasis
MMP: matrix metalloproteinase
SGRh2: 20(S)-ginsenoside Rh2
